# Virology analysis in HCV genotype 1-infected patients treated with the combination of simeprevir and TMC647055/ritonavir, with and without ribavirin, and JNJ-56914845

**DOI:** 10.1186/s12985-017-0760-2

**Published:** 2017-05-31

**Authors:** Leen Vijgen, Kim Thys, An Vandebosch, Pieter Van Remoortere, René Verloes, Sandra De Meyer

**Affiliations:** Janssen Research & Development, Janssen Pharmaceutica NV, Turnhoutseweg 30, 2340 Beerse, Belgium

**Keywords:** Hepatitis C virus, Genotype 1, Simeprevir, TMC647055/ritonavir, JNJ-56914845

## Abstract

**Background:**

In study TMC647055HPC2001, a 3-direct-acting-antiviral (DAA) regimen combining NS3/4A protease inhibitor simeprevir (SMV), non-nucleoside NS5B inhibitor TMC647055/ritonavir (RTV) and NS5A inhibitor JNJ-56914845 resulted in high sustained virologic response 12 weeks after actual end of treatment (SVR12) in chronic hepatitis C virus (HCV) genotype 1-infected patients. SVR12 rates were generally lower in the 2-DAA regimen SMV + TMC647055/RTV with or without ribavirin. The objective of this study was to identify and characterise pre-existing and emerging resistance-associated variants (RAVs) in patients enrolled in study TMC647055HPC2001.

**Methods:**

HCV population sequencing analyses were performed on baseline isolates from all patients (*n* = 90) and post-baseline isolates from patients with virologic failure (*n* = 22). In addition, deep sequencing and phenotypic analyses were performed on selected baseline and post-baseline isolates.

**Results:**

The majority of patients with virologic failure had emerging RAVs to all study drugs at the time of failure: in all 22 patients SMV RAVs emerged at NS3 positions 80, 155, 156 and/or 168, consistent with the known SMV resistance profile. Emerging TMC647055 RAVs at NS5B position 495 were detected in the majority of patients (16/22), and all 5 patients who failed the 3-DAA regimen had emerging JNJ-56914845 RAVs at NS5A positions 30 and/or 31. While at the end of study emerging SMV and TMC647055 RAVs were no longer observed by population sequencing in 40% (8/20) and 62.5% (10/16) of patients with follow-up data available, respectively, emerging JNJ-56914845 RAVs were still detected in all (5/5) patients.

**Conclusions:**

Virologic failure in the 2- and 3-DAA combinations was, in the majority of patients, associated with the emergence of RAVs to all study drugs. While emerging SMV and TMC647055 RAVs became undetectable during follow-up, JNJ-56914845 RAVs in NS5A were still observed at end of study.

**Trial registration number:**

NCT01724086 (date of registration: September 26, 2012)

**Electronic supplementary material:**

The online version of this article (doi:10.1186/s12985-017-0760-2) contains supplementary material, which is available to authorized users.

## Background

Hepatitis C virus (HCV) infection is a leading cause of chronic liver disease with an estimated 130–150 million people infected worldwide [1]. Since the approval in 2011 of the first direct-acting antivirals (DAAs), current therapy includes well-tolerated interferon (IFN)-free DAA combination regimens with high efficacy rates [2, 3]. For some regimens, the addition of ribavirin is indicated in certain patient populations to increase efficacy rates [4].

In the Phase 2a study TMC647055HPC2001 (NCT01724086), a 12-week 3-DAA regimen of simeprevir (SMV), TMC647055/ritonavir (RTV) and JNJ-56914845 resulted in high sustained virologic response 12 weeks after actual end of treatment (SVR12; 93% for HCV genotype [GT]1a- and 100% for GT1b-infected patients in the JNJ-56914845 60 mg group) while SVR12 rates were lower in the 12-week 2-DAA regimens of SMV and TMC647055/RTV with or without ribavirin (RBV) (SVR12 33 − 86% depending on HCV geno/subtype, presence of RBV and TMC647055 dose) [5].

SMV is a once-daily (QD), oral HCV NS3/4A protease inhibitor, approved with pegylated IFN (pegIFN)/RBV for the treatment of HCV GT1 and GT4 infection in the United States (US) and European Union (EU), and in IFN-free combination with sofosbuvir for GT1 infection in the US and GT1 and GT4 infection in the EU. The majority of HCV GT1-infected patients who failed SMV/pegIFN/RBV treatment in a Phase 2b/3 clinical trial had emerging mutations at NS3 positions 80, 122, 155 and/or 168, conferring high-level resistance to SMV in vitro [6]. Similar SMV high-level resistance mutations were observed in GT4-infected patients who failed SMV/pegIFN/RBV treatment and in GT1-infected patients with virologic failure upon 12 or 24 weeks’ SMV/sofosbuvir treatment [7–10]. At the end of study, these mutations could no longer be detected in half of the patients.

TMC647055 is an oral non-nucleoside inhibitor (NNI) binding at the thumb-1 NNI-1 site of the HCV NS5B polymerase [11]. The antiviral activity of TMC647055 in HCV GT1-infected patients is dose-dependent, with a median maximum decrease from baseline in HCV RNA of 2.4 log_10_ IU/mL and 3.4 log_10_ IU/mL at 1000 mg twice daily (BID) for 5 days in GT1a- (*n* = 3) and GT1b-infected (*n* = 3) patients, respectively [12]. Emerging TMC647055 resistance-associated variants (RAVs) at NS5B position 495 were observed in two GT1b-infected patients. Combination of TMC647055 (1000 mg BID) with SMV (150 mg QD) for 10 days in GT1-infected patients (seven GT1a and one GT1b) led to potent antiviral activity with a 4.64 log_10_ IU/mL median decrease in HCV RNA from baseline at day 11 with no viral breakthrough observed [13]. RTV was used as a pharmacokinetic enhancer to counteract cytochrome P450 3A4 induction by TMC647055, therefore boosting circulating plasma concentrations of TMC647055 and SMV when given in combination in study TMC647055HPC2001.

JNJ-56914845 (previously GSK2336805) is an oral inhibitor of HCV replication targeting the NS5A replication complex [14]. Single-dose administration of JNJ-56914845 to HCV GT1-infected patients (15 GT1a; two GT1b) resulted in rapid reductions in HCV RNA, with a mean decrease in HCV RNA from baseline within the first 24 hours of 2.18 log_10_ IU/mL for 30 mg (one GT1a) and 2.84 log_10_ IU/mL for 60 mg (four GT1a; one GT1b) [15]. Emerging JNJ-56914845 RAVs were detected in 13/23 patients at NS5A positions 28, 30, 31, 32 and/or 93.

The objective of this study was to identify and characterise pre-existing and emerging RAVs in patients enrolled in study TMC647055HPC2001.

## Methods

### Study design

**Fig. 1:**
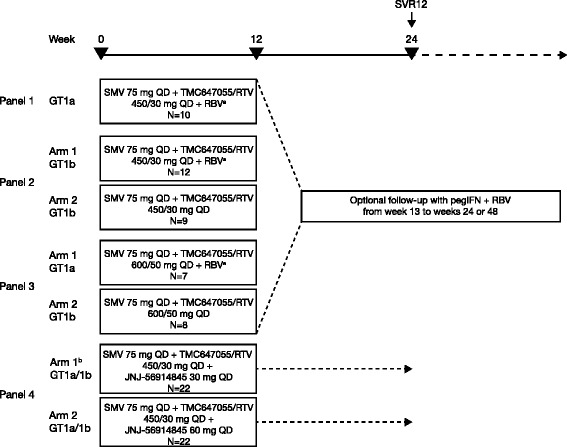
TMC647055HPC2001 study design. ^a^Ribavirin given BID at doses of 1000–1200 mg. ^b^Panel 4 Arm 1 included 1 GT1c- and 1 GT1l-infected patient, based on NS5B-based geno/subtyping. Follow-up therapy with pegIFN + RBV was based on on-treatment response and was initiated only if: week 4 HCV RNA ≥25 IU/mL (Panels 1–3: 36 weeks of follow-up therapy); week 4 HCV RNA <25 IU/mL detectable or HCV RNA confirmed detectable between week 4 and week 11 (Panels 1–2: 12 weeks of follow-up therapy). BID: twice daily; GT: genotype; pegIFN: pegylated interferon; QD: once daily; RBV: ribavirin; RTV: ritonavir; SMV: simeprevir; SVR12: sustained virologic response 12 weeks after actual end of treatment

The TMC647055HPC2001 study design is shown in Fig. 1 [5].

### HCV geno/subtype determination

HCV geno/subtypes were determined at screening by the VERSANT® HCV Genotype 2.0 (LiPA v2.0) or, if failed, by the Trugene HCV Genotyping assay (Siemens Healthcare Diagnostics, IL, USA). HCV geno/subtypes were determined pretreatment by sequencing an NS5B 329-bp region followed by basic local alignment search tool analysis. The results of the NS5B-based assay or, if missing, from the LiPA v2.0 or Trugene assay were used for virology analyses.

### HCV NS3/4A, NS5B and NS5A sequence analysis

The NS3/4A region or the NS3 protease, NS5B and NS5A gene were sequenced using population sequencing in baseline samples from all patients and post-baseline samples from patients with virologic failure [16] (see Additional file 1). In addition, Illumina deep sequencing was performed for a selection of samples (from Panels 1–2 and Panel 4), as described earlier [17]. Deep sequencing analysis was performed using a detection threshold of 1%. Sequencing resulted in an average coverage of 19,572 reads (range 7,177–36,508) per amino acid position in NS3 (amino acid position 1–181), of 20,594 reads (range 463–78,381) per amino acid position in NS5B, and of 23,045 reads (range 2,563–78,342) per amino acid position in NS5A. Baseline polymorphisms were defined as amino acid changes from the H77 (GenBank accession number AF009606) or the HCV Con1 (GenBank accession number AJ238799) reference sequences for HCV GT1a and GT1b, respectively. Emerging mutations were defined as amino acid changes from patient-specific baseline sequences based on population sequencing. RAVs were defined as amino acid substitutions with a fold change (FC) in 50% effective concentration (EC_50_) >2.0 for the respective drug, when tested as a site-directed mutant (SDM) in a transient replicon assay.

### Phenotypic characterisation using a transient replicon assay

SDMs were engineered in a GT1a or GT1b replicon. For the chimeric replicon assay, sequences of the NS3 protease domain (aa7-192), NS5B polymerase (full length) or NS5A (full length) from patient isolates were introduced in a GT1b replicon backbone generating chimeric replicons [18–21]. The NS5A chimeric replicon assay was performed at Monogram Bioscience Inc., LabCorp, San Francisco, CA, USA. Antiviral activity of the inhibitors against the NS3 protease and NS5B chimeric replicons was assessed in a transient replicon assay using luciferase read-out, as described earlier [22]. EC_50_ values were determined and compared with the EC_50_ of a reference GT1b wild-type replicon to calculate the FC in EC_50_ values.

### Ethical approval

The study was approved by the Institutional Review Board or Independent Ethics Committee at each participating centre, and met the ethical principles of the Declaration of Helsinki and Good Clinical Practice guidelines. All patients provided written, informed consent.

## Results

### Baseline polymorphisms

**Fig. 2:**
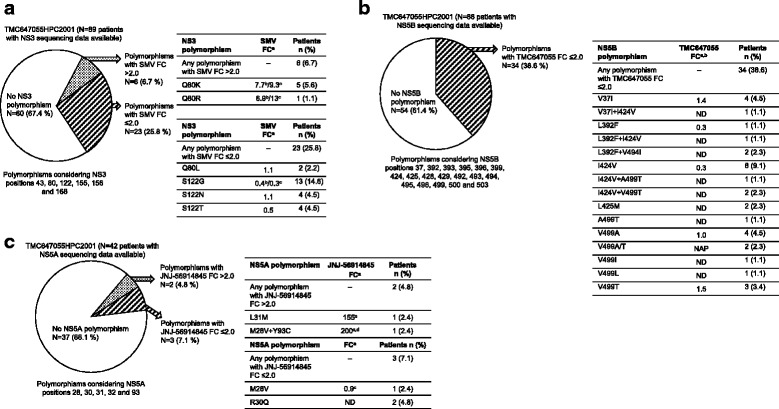
Prevalence of NS3 (**a**), NS5B (**b**) and NS5A (**c**) baseline polymorphisms in study TMC647055HPC2001. ^a^FC in EC_50_ values compared with GT1b wild-type replicon assessed as SDM in a transient replicon assay. ^b^SDM in GT1b replicon backbone. ^c^SDM in GT1a replicon backbone. ^d^SDM data for single Y93C. Baseline NS3 sequencing data were not available for 1 patient (HCV GT1l-infected patient); baseline NS5B and NS5A sequencing data were not available for 2 of the evaluable patients (HCV GT1l-infected patient and HCV GT1c-infected patient). EC_50_: 50% effective concentration; FC: fold change; GT: genotype; NAP: not applicable; ND: not determined; SDM: site-directed mutant; SMV: simeprevir

At baseline, SMV RAVs were observed in 6/89 GT1-infected patients (6.7%) with NS3 sequencing data available. These included Q80K (in 5/89 GT1 [5.6%]; 4/44 GT1a [9.1%]; 1/44 GT1b [2.3%]) and Q80R (in 1/89 GT1 [1.1%]) (Fig. 2). Two out of the four GT1a-infected patients with baseline Q80K were treated with the 2-DAA regimen in Panel 1 and did not achieve SVR. The two other GT1a-infected patients with baseline Q80K received the 3-DAA regimen in Panel 4; one patient achieved SVR while the other experienced viral relapse. The GT1b-infected patient with Q80R at baseline had detectable HCV RNA at the end of treatment and did not achieve SVR. TMC647055 RAVs were not observed at baseline. In Panel 4, JNJ-56914845 RAVs were detected in 2/42 (4.8%) GT1-infected patients with NS5A sequencing data available; L31M and Y93C (in combination with M28V), respectively (both in 1/27 GT1a [3.7%]). Both patients with baseline JNJ-56914845 RAVs achieved SVR.

**Fig. 3:**
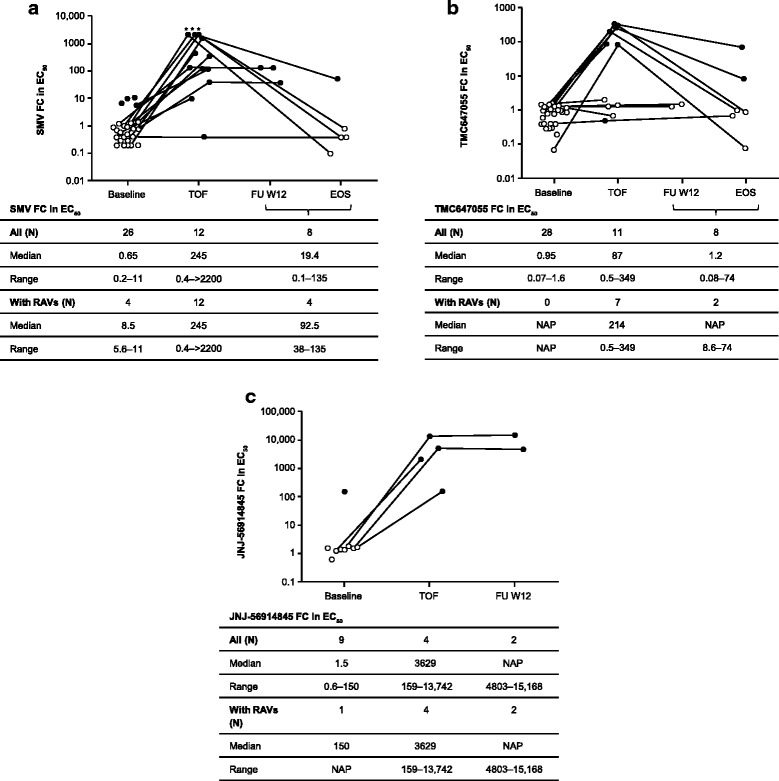
In vitro activity of SMV (**a**), TMC647055 (**b**) and JNJ-56914845 (**c**) against chimeric replicons containing the NS3 protease, NS5B polymerase or NS5A sequences, respectively, from isolates obtained at baseline, time of failure (TOF) and end of study (EOS) or follow-up week 12 (FU W12). Patients with RAVs, as detected by population sequencing in the corresponding plasma samples, are indicated with filled circles; patients without RAVs are indicated with open circles; for three samples at TOF, indicated with an asterisk, the SMV EC_50_ was above the highest test concentration with a censored FC in EC_50_ (i.e. >2200); the two isolates with SMV or TMC647055 RAVs detected in the plasma samples at TOF and wild-type sensitivity to SMV and TMC647055, respectively, did not contain these RAVs in the respective sequences included in the chimeric replicons. EC_50_: 50% effective concentration; EOS: end of study, corresponding to the sample from the last available time point in the study; FC: fold change; FU W12: follow-up week 12, corresponding to the sample obtained at 12 weeks after end of treatment; NAP: not applicable; RAV: resistance-associated variant; SMV: simeprevir; TOF: time of failure, corresponding to the sample obtained at TOF

In the phenotypic analysis of a subset of baseline isolates (Fig. 3), low-level resistance to SMV was observed for four GT1a isolates with SMV RAV Q80K (median FC in EC_50_ = 8.5; range 5.6–11). All baseline isolates remained fully susceptible to TMC647055 (FC in EC_50_ ≤ 2.0). One GT1a baseline isolate with NS5A RAV L31M displayed a 150-fold reduction in susceptibility to JNJ-56914845. For the baseline isolate with NS5A Y93C (+M28V), which was present as mixture with wild-type, the FC in EC_50_ was ≤2.0.

### Emergence of RAVs

Emergence of RAVs was investigated using population sequencing in the 22 patients with virologic failure (viral breakthrough, detectable HCV RNA at end of treatment [without viral breakthrough] or viral relapse [before or after follow-up week 12]) (Table 1).

**Table 1:** Population sequencing and deep sequencing data for patients with virologic failure

Pt	GT	Reason for failure	Gene	Baseline^a^		Time of failure^a^		End of study^a^	
				PS	DS	PS^b^	DS	PS^b^	DS
Panel 1									
1	GT1a	Viral breakthrough	NS3	None	None	**D168V**	D168F 48.7%	**R155K**	R155K 7.8%
							D168V 50.4%		D168E 7.1%
			NS5B	None	None	**P495L**	P495A 99.3%	**P495L**	P495L 29.0%
2	GT1a	Viral breakthrough	NS3	None	S122G 1.7%	**D168V**	Q80R 60.8%	**R155K**	S122T 2.5%
							R155K 61.6%		R155K 98.9%
							D168V 37.4%		
			NS5B	None	None	**P495L**	P495L 99.5%	None	P495L 13.6%
3	GT1a	Viral breakthrough	NS3	None	NA	**Q80R** + **R155K**	NA	**R155K**	NA
			NS5B	I424V + A499T	NA	I424V + A499T + **P495L**	NA	I424V + A499T	NA
4	GT1a	Viral breakthrough	NS3	Q80K	Q80K 99.1%	Q80K + **R155K**	NA	Q80K	Q80K 99.2%
			NS5B	L392F	L392F 99.7%	L392F/L + **P495L**	NA	L392F	L392F 99.4%
									P495L 1.4%
5	GT1a	Viral breakthrough	NS3	Q80K	Q80K 98.6%	Q80K + **R155K**	NA	Q80K + **D168E**	Q80K 99.4%
									R155K 74.2%
									D168E 24.6%
			NS5B	V37I	V37I 99.4%	V37I + **P495L/S**	NA	V37I + **P495A**	V37I 98.2%
									P495A 32.5%
6	GT1a	Late viral relapse^c^	NS3	None	None	**D168A**	ND	ND	ND
			NS5B	None	A499T 1.6%	None	ND	ND	ND
Panel 2									
7	GT1b	Viral breakthrough	NS3	S122T	S122T 92.8%	S122T + **D168V**	NA	S122T	S122T 99.1%
			NS5B	None	None	**P495L**	NA	None	None
8	GT1b	Viral breakthrough	NS3	None	None	**D168A/V**	D168A 63.6%	None	None
							D168T 2.2%		
							D168V 33.3%		
			NS5B	None	None	**P495L**	P495L 78.3%	**P495L**	P495L 99.0%
							P495S 20.0%		
9	GT1b	Detectable at EOT	NS3	None	None	**A156V**	A156V 99.8%	None	None
			NS5B	None	None	**P495L**	P495L 79.1%	None	None
10	GT1b	Viral relapse	NS3	None	ND	**D168T**	NA	None	None
			NS5B	V499A	ND	V499A	NA	V499A	V499A 99.4%
11	GT1b	Viral relapse	NS3	None	None	**D168V**	D168V 99.8%	None	None
			NS5B	None	None	**P495S**	P495S 99.5%	None	None
Panel 3									
12	GT1a	Detectable at EOT	NS3	None	ND	**Q80R** **+** **R155K**	ND	**Q80R**	ND
			NS5B	None	ND	**P495L**	ND	None	ND
13	GT1b	Viral breakthrough	NS3	None	ND	**D168V**	ND	**D168V**	ND
			NS5B	None	ND	**P495S**	ND	None	ND
14	GT1b	Detectable at EOT	NS3	Q80R	ND	Q80R + **D168E**	ND	Q80R + **D168E**	ND
			NS5B	I424V + V499T	ND	I424V + V499T + **P495S**	ND	I424V + V499T + **P495S**	ND
15	GT1b	Viral relapse	NS3	None	ND	**D168V**	ND	**D168V**	ND
			NS5B	V37I+ I424V	ND	V37I+ I424V + **P495L**	ND	V37I+ I424V + **P495L**	ND
16	GT1b	Viral relapse	NS3	S122T	ND	S122T + **D168V**	ND	S122T	ND
			NS5B	None	ND	**P495L**	ND	None	ND
17	GT1b	Late viral relapse^c^	NS3	None	ND	**D168A**	ND	ND	ND
			NS5B	V37I	ND	V37I	ND	ND	ND
Panel 4									
18	GT1a	Viral breakthrough	NS3	None	None	**R155K**	Q80R 9.3%	**R155K**	R155K 79.6%
							R155K 99.5%		
			NS5B	I424V	L392F 3.6%	I424V + **P495L**	I424L 2.5%	I424V	I424V 81.7%
					I424V 98.3%		I424V 92.2%		V494A 2.1%
							P495L 85.2%		
			NS5A	None	M28V 31.3%	**Q30E**	Q30E 99.2%	**Q30E**	Q30E 99.3%
19	GT1a	Viral relapse	NS3	None	None	**Q80R**	Q80R 99.0%	**Q80R**	Q80R 99.7%
			NS5B	None	None	None	None	None	None
			NS5A	None	None	**Q30R**	Q30R 99.1%	**Q30R**	Q30R 99.6%
20	GT1a	Viral relapse	NS3	S122T	S122T 19.6%	S122T + **R155K**	S122T 99.7%	S122T + **R155K**	S122T 98.0%
							R155K 99.1%		R155K 95.5%
			NS5B	None	None	None	None	None	None
			NS5A	None	None	**Q30H** **+** **L31M**	Q30E 2.0%	**Q30H** **+** **L31M**	Q30H 98.3%
							Q30H 93.6%		L31M 98.6%
							L31M 94.1%		
21^d^	GT1a	Viral relapse	NS3	None	None	**R155K**	R155K 99.4%	**R155K**	R155K 99.5%
			NS5B	None	None	None	None	None	None
			NS5A	None	None	**Q30E**	Q30E 99.0%	**Q30E**	Q30E 99.5%
22	GT1a	Viral relapse	NS3	Q80K	Q80K 97.7%	Q80K + **R155S**	Q80K 99.8%	Q80K	Q80K 97.0%
							R155S 99.8%		R155S 8.2%
			NS5B	None	None	**P495L**	P495L 99.6%	**P495L**	P495L 85.9%
			NS5A	M28V	M28V 23.3%	**L31M**	L31M 99.7%	**L31M**	M28A 9.4%
									L31M 87.0%

^a^Amino acid substitutions are described considering six NS3 positions of interest (43, 80, 122, 155, 156 and 168), 18 NS5B positions of interest (37, 392, 393, 395, 396, 399, 424, 425, 428, 429, 492, 493, 494, 495, 496, 499, 500 and 503) and five NS5A positions of interest (28, 30, 31, 32 and 93). RAVs (i.e. amino acid substitutions, when introduced as SDM in a GT1a or GT1b replicon, associated with a fold change in EC_50_ > 2.0) are underlined. Absence of amino acid substitutions considering the positions of interest is indicated with ‘none’. DS data are reported when available for all genes of interest at a specific time point. ^b^Amino acid substitutions shown in bold indicate emerging amino acid substitutions compared to baseline, based on PS. ^c^Late viral relapse defined as subject with SVR12 but with HCV RNA ≥25 IU/mL at follow-up week 24 visit, the last scheduled visit in the study. ^d^For subject 21, end of study DS data were not available, instead data from the follow-up week 12 visit are shown

*DS* deep sequencing, *EC*_*50*_ 50% effective concentration, *EOT* end of treatment, *GT* genotype, *HCV* hepatitis C virus, *NA* not available (no DS data available due to failure of amplification or Illumina DS reaction), *ND* not determined, *PS* population sequencing, *Pt* patient, *RAV* resistance-associated variant, *SDM* site-directed mutant, *SVR12* sustained virologic response 12 weeks after actual end of treatment

In 14/17 patients (82.4%) with virologic failure in the 2-DAA regimen, emerging RAVs to both SMV and TMC647055 were detected at time of failure. SMV RAVs at NS3 positions 80, 155, 156 and/or 168 were observed in all 17 patients. The majority (9/10) of GT1b-infected patients had an emerging mutation at NS3 position 168, while either an emerging R155K (alone or in combination with a Q80R) (in 4/7) or an emerging mutation at NS3 position 168 (in 3/7) was observed in the GT1a-infected patients. Emerging TMC647055 RAVs at NS5B position 495 were detected in 14/17 patients, and included mainly P495L (in 11/14).

In all five patients with virologic failure in the 3-DAA regimen (all GT1a), emerging RAVs to both SMV and JNJ-56914845 were observed at time of failure. SMV RAVs emerged at NS3 positions 80 or 155; emerging JNJ-56914845 RAVs were detected at NS5A positions 30 and/or 31 (Q30E [*n* = 2], Q30R [*n* = 1], L31M [*n* = 1] and Q30H + L31M [*n* = 1]). Emerging TMC647055 RAVs were observed in 2/5 patients, and involved P495L in both.

Illumina deep sequencing data were available for a subset of time of failure isolates (Table 1). In the three patients in Panel 4 without emerging TMC647055 RAVs at time of failure based on population sequencing, no RAVs could be detected by deep sequencing. Additional RAVs at a read frequency ≤25% were detected by deep sequencing in three patients with RAVs detected by population sequencing (patients 8, 18, 20). Deep sequencing identified additional emerging RAVs at a read frequency >25% in two patients at time of failure (patients 1, 2). RAVs emerging at time of failure were not detected at baseline by deep sequencing in the set of samples analysed.

For all three inhibitors, a reduction in in vitro activity was observed against time of failure isolates that contained SMV, TMC647055 or JNJ-56914845 RAVs (Fig. 3). Isolates collected at time of failure, with no TMC647055 RAVs detected, remained fully susceptible to TMC647055 (FC in EC_50_ ≤ 2.0).

### Persistence of emerging RAVs

In 8/20 patients (40%) with emerging SMV RAVs at time of failure and follow-up NS3 sequencing data available, these SMV RAVs were no longer observed by population sequencing at end of study (Table 1). The median time between time of failure and end of study NS3 sequence was 20.6 weeks (range: 4.7–34.1 weeks) and 25.5 weeks (range: 4.1–28.1 weeks), respectively, for the eight patients without and 12 patients with emerging SMV RAVs detected at end of study. Ten out of 16 patients (62.5%) with emerging TMC647055 RAVs at time of failure and follow-up NS5B sequencing data available had lost these mutations at end of study, as assessed by population sequencing. The median time between time of failure and end of study NS5B sequence was 25.8 weeks (range: 20.1–28.4 weeks) and 23.9 weeks (range: 4.1–34.1 weeks), respectively, for the 10 patients without and the six patients with emerging TMC647055 RAVs detected at end of study. For all five patients with emerging JNJ-56914845 RAVs at time of failure, these RAVs were still observed at end of study, with a median time between time of failure and end of study NS5A sequence of 20.1 weeks (range: 0–26.6 weeks). In the majority of patients with emerging RAVs no longer observed at end of study by population sequencing, these were also not detected by deep sequencing. In four patients (patients 2, 4, 18, 21), emerging TMC647055 or SMV RAVs were still detected by deep sequencing (read frequency <25%), while no longer present based on population sequencing.

In vitro susceptibility to the respective drugs was reduced when SMV, TMC647055 or JNJ-56914845 RAVs were still observed by population sequencing at end of study or follow-up week 12 (in case end of study isolate was not tested) (Fig. 3). When these RAVs were no longer detected by population sequencing, including in two patients (patients 2 and 4) with TMC647055 RAVs still detected by deep sequencing (read frequency <25%), wild-type sensitivity to the respective drugs was found.

## Discussion

In this study, pre-existing RAVs were identified at low frequency. Baseline SMV RAVs were observed in 6.7% of patients with NS3 sequencing data available, and included Q80K in 5.6% of GT1-infected patients and in 9.1% of GT1a-infected patients. In the SMV/pegIFN/RBV Phase 2b/3 studies, the baseline prevalence of Q80K was higher (13.6% in GT1-infected patients; 29.5% in GT1a-infected patients), which can be explained by the fact that the global Phase 2b/3 studies included North America, a region with a high GT1a prevalence and a high Q80K prevalence within GT1a, while study TMC647055HPC2001 was performed in Europe [6]. In the SMV/pegIFN/RBV Phase 2b/3 studies, SVR rates were substantially reduced in GT1a-infected patients with baseline Q80K compared to those without this polymorphism [23–25]. The two GT1a-infected patients with baseline Q80K in the 2-DAA regimen did not achieve SVR; however, virologic failure rates were generally high (6/10) in Panel 1. In the 3-DAA regimen, one of the two GT1a-infected patients with baseline Q80K achieved SVR. Baseline JNJ-56914845 RAVs were present in 4.8% (2/42) of patients with NS5A sequencing data available; both patients achieved SVR. However, the low number of patients with baseline RAVs did not allow conclusions to be drawn on the impact of the presence of baseline RAVs on treatment outcome.

In the majority of patients with virologic failure, emerging RAVs to all study drugs were observed at time of failure. Similar findings of emerging resistance to multiple classes of DAAs have been described in patients with virologic failure in other 12-week combination regimens of DAAs with similar mechanisms of action i.e. an NS3/4A protease inhibitor, paritaprevir/ritonavir, combined with an NNI, dasabuvir, with or without an NS5A inhibitor, ombitasvir [26, 27]. All patients had SMV RAVs at time of failure, detected at NS3 positions 80, 155, 156 and/or 168, consistent with the previously characterised SMV resistance profile [6, 7, 9, 10, 28]. Emerging TMC647055 RAVs were found at time of failure in the majority of patients (in 14/17 and 2/5 patients with virologic failure in the 2- and 3-DAA regimens, respectively). These were observed at NS5B position 495 only, similar to the findings from in vitro TMC647055 selection experiments [11] and from the Phase 1b study that evaluated TMC647055 in monotherapy and in combination with SMV [12, 13]. There was no difference found in emerging RAVs between a low dose of TMC647055/RTV (450 mg QD; Panels 1–2) and a high dose (600 mg QD; Panel 3) or between HCV geno/subtypes. All five 3-DAA failure patients had emerging JNJ-56914845 RAVs at NS5A positions 30 and/or 31. RAVs at these positions were also identified in JNJ-56914845 in vitro selection experiments and in the Phase 1 monotherapy study [14, 15]. The RAVs observed at time of failure were associated with a >100-fold reduction in JNJ-56914845 in vitro activity [14]. No in vitro JNJ-56914845 susceptibility data were available for Q30E; however, from data available from other NS5A inhibitors, it can be expected that this mutation also confers resistance to JNJ-56914845 [29]. Illumina deep sequencing, performed for a subset of time of failure isolates, confirmed the absence of TMC647055 RAVs in patients without these RAVs detected by population sequencing. For the selected set of samples, none of the RAVs emerging at time of failure were found to be already present at baseline by using deep sequencing.

While emerging SMV and TMC647055 RAVs became undetectable by population sequencing at end of study in 40% (8/20) and 62.5% (10/16) of patients, respectively, emerging JNJ-56914845 RAVs were still observed at the end of study in all five 3-DAA failure patients. These observations confirm earlier findings of disappearance of emerging SMV RAVs in half of the patients at end of study in SMV/pegIFN/RBV studies [6] (as assessed by population sequencing), as well as the long-term persistence of emerging NS5A RAVs that has been demonstrated in patients who failed ledipasvir-containing regimens [30].

In the majority of patients in whom emerging RAVs in the NS3 and NS5B regions were no longer observed at end of study by population sequencing, these were also no longer detected by the more sensitive Illumina deep sequencing technology, in line with findings from previous studies [31, 32].

Phenotypic analysis showed a reduction in sensitivity to the study drugs when RAVs were detected by population sequencing. Consistency between the detection of SMV RAVs in clinical isolates and their in vitro susceptibility to SMV was also demonstrated in a recent study investigating clinical isolates of HCV GT1-infected patients enrolled in SMV Phase 1–3 clinical studies [20].

## Conclusions

Virologic failure in study TMC647055HPC2001 was associated with the emergence of RAVs to all study drugs in the majority of patients. Treatment-emergent SMV and TMC647055 RAVs became undetectable over time in many of the patients after treatment was stopped, while emerging JNJ-56914845 RAVs could still be detected at end of study in the five 3-DAA failure patients.

## Additional file

Additional file 1: Study design. HCV NS3/4A, NS5B and NS5A sequence analysis. (DOCX 32 kb)

## Abbreviations

BID: Twice daily
DAA: Direct-acting antiviral
EC_50_: 50% effective concentration
EU: European Union
FC: Fold change
GT: Genotype
HCV: Hepatitis C virus
IFN: Interferon
NAP: Not applicable
ND: Not determined
NNI: Non-nucleoside inhibitor
pegIFN: Pegylated interferon
QD: Once daily
RAV: Resistance-associated variant
RBV: Ribavirin
RTV: Ritonavir
SDM: Site-directed mutant
SMV: Simeprevir
SVR12: Sustained virologic response 12 weeks after actual end of treatment
US: United States
