# Supplementary material for: Virology analysis in HCV genotype 1-infected patients treated with the combination of simeprevir and TMC647055/ritonavir, with and without ribavirin, and JNJ-56914845
Source: Virol J. 2017 May 31;14:101. doi: 10.1186/s12985-017-0760-2 (PMC5452362; doi:10.1186/s12985-017-0760-2)
Supplement: Additional file 1: — Study design. HCV NS3/4A, NS5B and NS5A sequence analysis. (DOCX 32 kb) [file 12985_2017_760_MOESM1_ESM.docx]

Additional Material

Virology analysis in HCV genotype 1-infected patients treated with the combination of simeprevir and TMC647055/ritonavir, with and without ribavirin, and JNJ-56914845

Leen Vijgen, Kim Thys, An Vandebosch, Pieter Van Remoortere, René Verloes, Sandra De Meyer

# Study design

## HCV NS3/4A, NS5B and NS5A sequence analysis

Hepatitis C virus (HCV) NS3/4A, NS5B and NS5A (Panel 4 only) sequencing was performed at baseline for all patients, and at post-baseline for patients with virologic failure, based on the time point of failure, availability of samples until the end of study and the sensitivity of the sequencing assay. Lists of amino acid positions of interest in NS3, NS5B and NS5A were defined to guide the analysis. Two lists of NS3 amino acid positions of interest were defined to guide the analyses. The first list comprised six NS3 amino acid positions: 43, 80, 122, 155, 156 and 168; specific amino acid changes at one or more of these positions are known to confer reduced susceptibility to simeprevir *in vitro* [1]. The second list of 18 NS3 amino acid positions also included NS3 positions that have been associated with resistance to other HCV NS3/4A protease inhibitors or that were considered of interest based on observations in *in vitro* or *in vivo* studies with simeprevir; this list comprised positions: 36, 41, 43, 54, 55, 80, 107, 122, 132, 138, 155, 156, 158, 168, 169, 170, 174 and 175 [1,2].

The list of NS5B amino acid positions of interest was defined based on the NS5B binding pocket residues for non-nucleoside inhibitors (NNIs) binding in the NNI-1 pocket of the NS5B polymerase, and this list comprised 18 NS5B positions: 37, 392, 393, 395, 396, 399, 424, 425, 428, 429, 492, 493, 494, 495, 496, 499, 500 and 503 [3]. One or more changes at a number of these positions have emerged in TMC647055 *in vitro* selection experiments, conferring reduced susceptibility [4].

Similarly to NS3, two lists of NS5A amino acid positions were defined to guide the analysis. The first list included 5 NS5A amino acid positions, i.e. 28, 30, 31, 32 and 93, since specific amino acid changes at these positions have been found to cause a decrease in *in vitro* activity of JNJ-56914845 [5]. The second list also included NS5A positions that have been associated with resistance to other HCV NS5A inhibitors, and consisted of 14 NS5A positions: 23, 24, 28, 30, 31, 32, 38, 54, 56, 58, 62, 64, 92 and 93 [6].

# References

1. Lenz O, Verbinnen T, Lin T-I, Vijgen L, Cummings MD, Lindberg J, et al. *In vitro* resistance profile of the hepatitis C virus NS3/4A protease inhibitor TMC435. Antimicrob Agents Chemother. 2010;54:1878-87.

2. Lontok E, Harrington P, Howe A, Kieffer T, Lennerstrand J, Lenz O, et al. Hepatitis C virus drug resistance-associated substitutions: State of the art summary. Hepatology. 2015;62:1623-32.

3. Pauwels F, Mostmans W, Quirynen LM, van der Helm L, Boutton CW, Rueff AS, et al. Binding-site identification and genotypic profiling of hepatitis C virus polymerase inhibitors. J Virol. 2007;81:6909-19.

4. Devogelaere B, Berke JM, Vijgen L, Dehertogh P, Fransen E, Cleiren E, et al. TMC647055, a potent nonnucleoside hepatitis C virus NS5B polymerase inhibitor with cross-genotypic coverage. Antimicrob Agents Chemother. 2012;56:4676-84.

5. Walker J, Crosby R, Wang A, Woldu E, Vamathevan J, Voitenleitner C, et al. Preclinical characterization of GSK2336805, a novel inhibitor of hepatitis C virus replication that selects for resistance in NS5A. Antimicrob Agents Chemother. 2014;58:38-47.

6. Sarrazin C. The importance of resistance to direct antiviral drugs in HCV infection in clinical practice. J Hepatol. 2016;64:486-504.
